# Bempedoic Acid in Patients with Chronic Coronary Syndrome Not Achieving LDL Targets Despite Intensive Therapy: A Real-World Study from Spain

**DOI:** 10.3390/jcm15156102

**Published:** 2026-08-05

**Authors:** José Javier Gómez-Barrado, Paula Gómez-Turégano, Miguel Turégano-Yedro, Elena Jiménez-Baena, Ana Isabel Fernández-Chamorro, Marta Gómez-Turégano

**Affiliations:** 1 Department of Cardiology, Hospital Universitario San Pedro de Alcántara, 10003 Cáceres, Spain; paula.gt@hotmail.es (P.G.-T.); tureyedro@gmail.com (M.T.-Y.); elena.jimenezb@salud-juntaex.es (E.J.-B.); anabel1913@hotmail.com (A.I.F.-C.); martagturegano@gmail.com (M.G.-T.); 2 Faculty of Medicine and Health Sciences Biomedical Sciences, University of Extremadura, 06006 Badajoz, Spain; 3 Primary Care Department, Centro de Salud “El Casar de Cáceres”, Cáceres Healthcare Area, 10190 Cáceres, Spain

**Keywords:** bempedoic acid, ATP citrate lyase, low-density lipoprotein cholesterol, chronic coronary syndrome, lipid-lowering therapy, lipoprotein(a), cardiovascular risk, real-world study

## Abstract

**Background/Objectives:** Achieving guideline-recommended low-density lipoprotein cholesterol (LDL-C) targets in patients with chronic coronary syndrome (CCS) remains challenging despite intensive lipid-lowering therapy. Bempedoic acid (BA) offers an oral therapeutic option, although data on its clinical performance and patient-level determinants of response in real-world Spanish settings are limited. **Methods:** We conducted a prospective multicentre study across the four healthcare areas of Cáceres province, Spain, including consecutive CCS patients with LDL-C ≥ 55 mg/dL despite stable intensive lipid-lowering therapy. BA 180 mg/day was added to background treatment. Lipid parameters, metabolic profile, and safety outcomes were assessed after a median follow-up of 28 weeks (IQR 23–47). Multivariable analyses were performed to identify factors associated with of LDL-C reduction and target attainment. A total of 118 patients were analyzed for outcomes. **Results:** A total of 118 patients (mean age 62.4 ± 10.0 years; 79.2% male) were included. BA reduced LDL-C by 22.8% (−16.36 mg/dL; *p* < 0.001), enabling 48.3% of patients to achieve LDL-C < 55 mg/dL. Higher baseline LDL-C (β = −0.515; *p* = 0.001) and the presence of diabetes mellitus (B = 13.8 mg/dL; *p* = 0.024) were independently associated with greater LDL-C reduction. Notably, 56.3% of patients presented with elevated baseline lipoprotein(a) levels (>50 mg/dL), describing a high underlying burden of residual risk. BA was well tolerated, with a modest increase in uric acid levels but no gout events and a high treatment persistence rate (94.4%). **Conclusions:** In a real-world CCS population receiving intensive lipid-lowering therapy, BA provides clinically meaningful LDL-C reduction with a favorable safety profile in this multicentre cohort from Cáceres province. Patients with higher baseline LDL-C and diabetes derive greater benefit, supporting a more personalized approach to therapy. These findings reinforce the role of BA in Spanish patients as an intermediate step in lipid-lowering strategies before escalation to more costly therapies, addressing the scarcity of local real-world data.

## 1. Introduction

Atherosclerotic cardiovascular disease (ASCVD) remains the leading cause of morbidity and mortality worldwide despite major advances in lipid-lowering therapies. Low-density lipoprotein cholesterol (LDL-C) is a causal and modifiable driver of atherogenesis, and cumulative LDL-C exposure is directly associated with cardiovascular risk [1,2]. Consequently, intensive LDL-C reduction represents a cornerstone of secondary prevention in patients with chronic coronary syndrome (CCS).

Current ESC/EAS guidelines recommend stringent LDL-C targets (<55 mg/dL and ≥50% reduction from baseline) in very-high-risk patients [3]. However, real-world data consistently demonstrate that a substantial proportion of patients fail to achieve these targets despite treatment with high-intensity statins and ezetimibe, reflecting suboptimal implementation of guideline-recommended therapies in routine clinical practice [4,5,6]. This gap is particularly relevant in Southern European populations, where therapeutic inertia, comorbidity burden, and limited access to advanced lipid-lowering therapies may contribute to suboptimal LDL-C control. Beyond traditional lipid parameters, the identification and monitoring of specific biomarkers have become crucial in the management of ASCVD. Recent evidence underscores that a comprehensive evaluation of inflammatory and metabolic biomarkers can better refine cardiovascular risk stratification and guide the intensification of lipid-lowering therapies [7]. In this context, BA not only addresses LDL-C targets but also influences other relevant markers of the atherogenic process.

In this context, there is a growing need for effective, safe, and accessible lipid-lowering strategies that can be readily implemented in daily clinical practice. Bempedoic acid (BA) is an oral prodrug that inhibits ATP citrate lyase (ACLY), a key enzyme upstream of HMG-CoA reductase, thereby reducing hepatic cholesterol synthesis and increasing LDL receptor expression [8,9]. Its liver-selective activation which may reduce skeletal muscle exposure and potentially improve tolerability, making it an attractive option for combination therapy, particularly in patients with statin intolerance or insufficient response.

Randomized clinical trials from the CLEAR program have demonstrated that BA significantly reduces LDL-C levels and improves cardiovascular outcomes, particularly in statin-intolerant individuals [10,11,12,13]. In addition, recent analyses have shown that the cardiovascular benefit of BA is proportional to the achieved LDL-C reduction and comparable to that observed with statins [14]. However, clinical trial populations may not fully reflect real-world complexity, where multimorbidity, adherence, and treatment heterogeneity influence therapeutic effectiveness.

Recent real-world studies have confirmed the effectiveness and tolerability of BA in routine clinical practice. In particular, a multicentre analysis from Southern Europe by Russo et al. demonstrated significant LDL-C reductions and high adherence rates in a heterogeneous population with dyslipidaemia [15]. Similarly, previous real-world experience in preventive cardiology settings has supported the clinical utility of BA [16]. However, these studies have primarily focused on overall treatment performance, with limited data on patient-level determinants of response and residual cardiovascular risk.

Furthermore, recent expert consensus documents emphasize the importance of stepwise combination lipid-lowering strategies, including the use of BA as an intermediate therapeutic option before escalation to PCSK9 inhibitors [17,18].

In addition to LDL-C, lipoprotein(a) [Lp(a)] represents an important contributor to residual cardiovascular risk. Elevated Lp(a) levels are largely genetically determined and are associated with increased atherosclerotic burden and cardiovascular events, with minimal response to conventional lipid-lowering therapies [19,20]. Therefore, patients with elevated Lp(a) may remain at high risk despite achieving LDL-C targets, highlighting the need for comprehensive risk assessment.

Accordingly, beyond evaluating the overall effectiveness of BA, there is a clinical need to identify which patients derive the greatest benefit from this therapy and how it may help address residual lipid-related risk in real-world settings.

The present study aims to provide robust, real-world evidence confirming the effectiveness and safety of BA in a multicentre cohort of Spanish patients with CCS receiving intensive lipid-lowering therapy. In addition, we sought to identify potential clinical predictors of LDL-C response and to characterize residual cardiovascular risk, including the burden of elevated Lp(a). By doing so, we aim to provide clinically relevant insights for personalized lipid-lowering strategies and to address the current scarcity of real-world data on BA in the Spanish population, strengthening the existing body of evidence with confirmatory data from routine clinical practice.

## 2. Materials and Methods

### 2.1. Study Design and Population

This was a prospective, multicentre, observational study conducted across the four healthcare areas of Cáceres province, Spain, with patients being seen at Hospital Universitario San Pedro de Alcántara as the coordinating centre. A total of 125 consecutive patients with CCS, aged ≥18 years, were included if they had LDL-C levels ≥ 55 mg/dL despite at least 8 weeks of stable intensive lipid-lowering therapy, defined as high-intensity statin plus ezetimibe, or a PCSK9 inhibitor plus ezetimibe in statin-intolerant patients. CCS was diagnosed according to the 2019 ESC Guidelines and included patients with documented coronary artery disease, defined by a previous myocardial infarction, prior coronary revascularization, or angiographically documented obstructive coronary artery disease in clinically stable patients [3]. To minimize confounding, all patients were required to maintain their baseline lipid-lowering therapy unchanged throughout the entire follow-up period. Among statin-tolerant patients, background therapy consisted of maximally tolerated high-intensity statin therapy (rosuvastatin 20 mg/day or atorvastatin 80 mg/day) plus ezetimibe 10 mg/day. Patients with documented statin intolerance received a PCSK9 monoclonal antibody plus ezetimibe, and no changes to background lipid-lowering therapy were allowed during follow-up.

PCSK9 inhibitors used in this cohort were exclusively monoclonal antibodies (mAbs), specifically alirocumab and evolocumab; no patients received inclisiran during the study period.

Exclusion criteria were age < 18 years, pregnancy or breastfeeding, known hypersensitivity to bempedoic acid, severe renal impairment (estimated glomerular filtration rate [eGFR] < 30 mL/min/1.73 m^2^), chronic liver disease, or active malignancy.

Statin intolerance was defined according to clinical criteria as the inability to tolerate at least two different statins (one at the lowest approved dose) due to adverse effects or laboratory abnormalities [17]. Cardiovascular risk was classified according to current ESC/EAS guidelines [3].

The study protocol was designed in late 2025 and was subsequently approved by the local ethics committee (Comité de Ética de la Investigación con Medicamentos, CEIM de Cáceres) in January 2026 (official certificate issued on 5 February 2026). Prospective patient recruitment and all clinical procedures were conducted strictly following this approval, between February 2026 and the end of the study period. All participants provided written informed consent prior to their inclusion in the study.

BA (180 mg once daily) was added to ongoing background therapy. Baseline data included cardiovascular risk factors, comorbidities, prior cardiovascular interventions, anthropometric parameters, and laboratory values (total cholesterol, LDL-C, HDL-C, triglycerides, creatinine, eGFR, uric acid, and fasting glucose).

Follow-up laboratory assessments were performed after at least 8 weeks of treatment, with a median follow-up duration of 28 weeks (IQR 23–47 weeks). Treatment adherence was assessed during follow-up clinical visits through patient interviews and verification of pharmacy dispensing records. Adverse events and treatment discontinuations were systematically recorded.

LDL-C targets were defined according to the current ESC/EAS guidelines (<55 mg/dL for very-high-risk patients) [3].

### 2.2. Definition of Lipid Variables

The following lipid parameters were defined:

Untreated baseline LDL-C: the earliest documented LDL-C measurement obtained prior to initiation of any lipid-lowering therapy.

Pre-BA LDL-C: LDL-C measurement obtained immediately before initiation of BA after at least 8 weeks of stable background lipid-lowering therapy.

LDL-C calculation: estimated using the Friedewald formula when triglyceride levels were <150 mg/dL; otherwise, a standardized direct enzymatic assay was used.

Non-HDL cholesterol was calculated as total cholesterol minus HDL cholesterol.

Lp(a) concentrations were determined by a particle-enhanced immunoturbidimetric assay (Tina-quant Lp(a) Gen.2, Roche Diagnostics). This methodology is isoform-independent, as it employs polyclonal antibodies directed against epitopes present in the apolipoprotein(a) particle regardless of its size. The assay is traceable to the IFCC reference material (SRM 2B), ensuring the clinical validity of the 50 mg/dL threshold used in this study. Baseline Lp(a) was measured to characterize the burden of genetically determined residual cardiovascular risk in this very-high-risk cohort. Since BA is not expected to substantially modify Lp(a) concentrations, serial measurements were not included in the study protocol, as assessment of treatment-related changes in Lp(a) was not a study objective.

### 2.3. Study Endpoints

The primary endpoints were:

Absolute and relative change in LDL-C levels

Proportion of patients achieving LDL-C < 55 mg/dL

Secondary endpoints included:

Changes in total cholesterol, non-HDL-C, HDL-C, triglycerides, uric acid, creatinine, and eGFR

Treatment tolerability and safety

Treatment discontinuation

Predictors of LDL-C reduction and achievement of LDL-C targets

Safety outcomes specifically included musculoskeletal symptoms, gastrointestinal adverse events, and hyperuricemia.

### 2.4. Statistical Analysis

Continuous variables are presented as mean ± standard deviation or median [interquartile range], depending on distribution, which was assessed using the Kolmogorov–Smirnov test.

Comparisons between baseline and follow-up values were performed using paired Student’s *t*-test or Wilcoxon signed-rank test, as appropriate. Categorical variables are expressed as counts and percentages and were compared using the χ^2^ test or Fisher’s exact test.

Comparisons between independent subgroups were conducted using the unpaired Student’s *t*-test or Mann–Whitney U test.

A two-sided *p*-value < 0.05 was considered statistically significant.

### 2.5. Regression Models

Multiple linear regression analysis was performed to identify independent factors associated with absolute LDL-C reduction. To ensure model robustness and avoid overfitting, only variables with *p* < 0.10 in univariable analyses or those with high clinical relevance were included. The final model was refined using a stepwise approach, ensuring an adequate ratio of subjects to independent variables. Multicollinearity was assessed using the variance inflation factor (VIF < 2.5).

Binary logistic regression analysis was used to identify factors associated with achieving LDL-C < 55 mg/dL using a forward stepwise approach. Model calibration was assessed using the Hosmer–Lemeshow test. Internal model stability and consistency were verified by ensuring the persistence of significant predictors across different selection methods and by confirming that model assumptions—including linearity, homoscedasticity, and normality of residuals—were fully met. Although internal validation techniques such as bootstrapping were not performed due to sample size constraints, model stability was assessed through consistency across selection methods and verification of assumptions. Given the sample size, the number of variables included in the final multivariable models was restricted to avoid overfitting, maintaining an appropriate subject-to-variable ratio. All analyses were performed using SPSS version 29 (IBM Corp., Armonk, NY, USA). During the preparation of this work, the authors used ChatGPT (GPT-4) for the purpose of literature search and bibliography compilation. The authors have since thoroughly reviewed, verified, and edited the final reference list against official databases, and assume full responsibility for the content and integrity of this publication.

## 3. Results

### 3.1. Study Population

**Figure 1:**
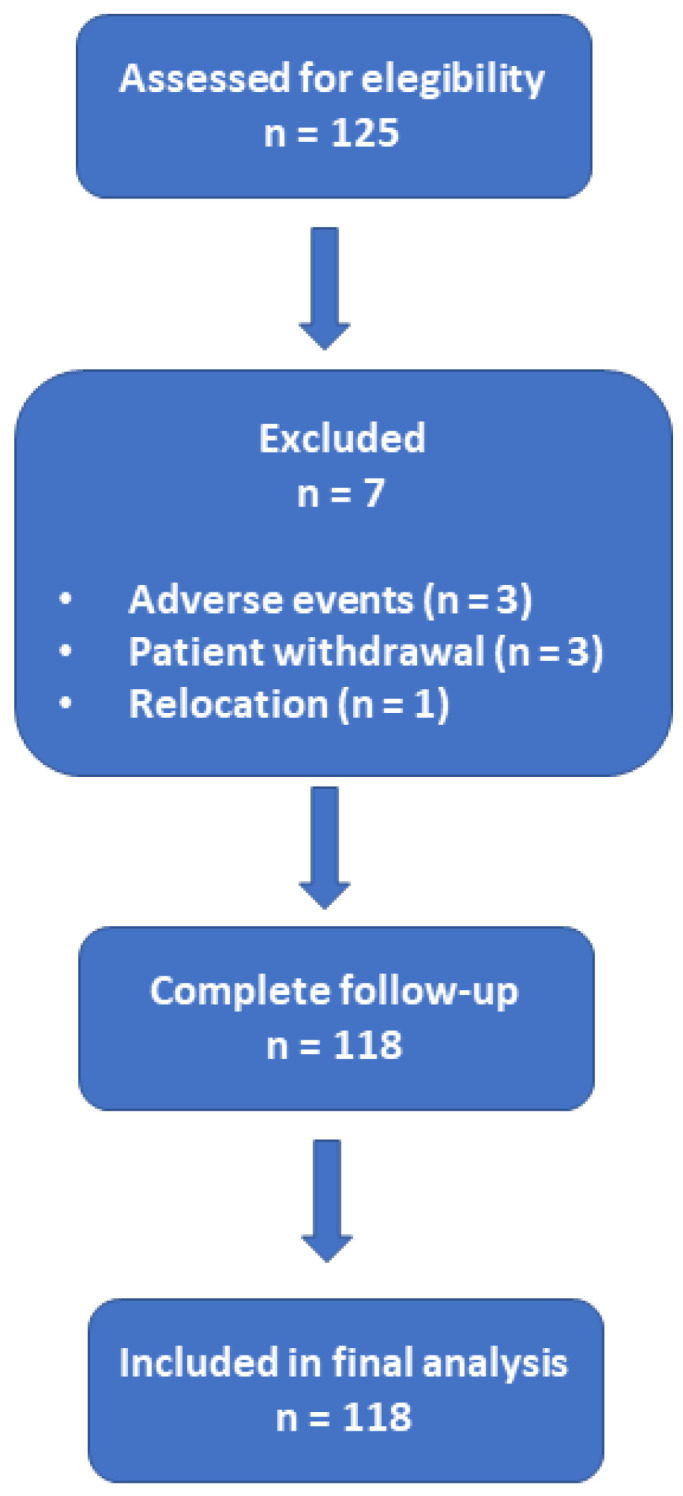
Flow diagram of patient selection. Flow diagram showing the number of patients screened, reasons for exclusion, number starting bempedoic acid, those with completed follow-up, and the final study population included in the analysis.

A total of 125 consecutive patients with CCS were initially enrolled. Seven patients were excluded due to treatment discontinuation or incomplete follow-up, resulting in a final study population of 118 patients (Figure 1).

The mean age was 62.4 ± 10.1 years, and 79.2% were male. Most patients were receiving intensive lipid-lowering therapy, consisting of high-intensity statin plus ezetimibe (*n* = 120), while a minority were treated with a PCSK9 inhibitor plus ezetimibe due to statin intolerance (*n* = 5). Baseline characteristics are summarized in Table 1.

Baseline characteristics are presented for all enrolled patients before follow-up. Efficacy and safety analyses were performed in the 118 patients who completed follow-up.

Data are presented as mean ± standard deviation (SD) or number (percentage), as appropriate. BMI: body mass index; ASCVD: atherosclerotic cardiovascular disease; eGFR: estimated glomerular filtration rate; PCI: percutaneous coronary intervention; CABG: coronary artery bypass grafting; PCSK9: proprotein convertase subtilisin/kexin type 9.

Patients were recruited from the four healthcare areas of Cáceres province, with Hospital Universitario San Pedro de Alcántara serving as the coordinating centre.

The study population was predominantly composed of very-high-risk patients according to ESC/EAS criteria.

### 3.2. Effects on Lipid Profile

**Figure 2:**
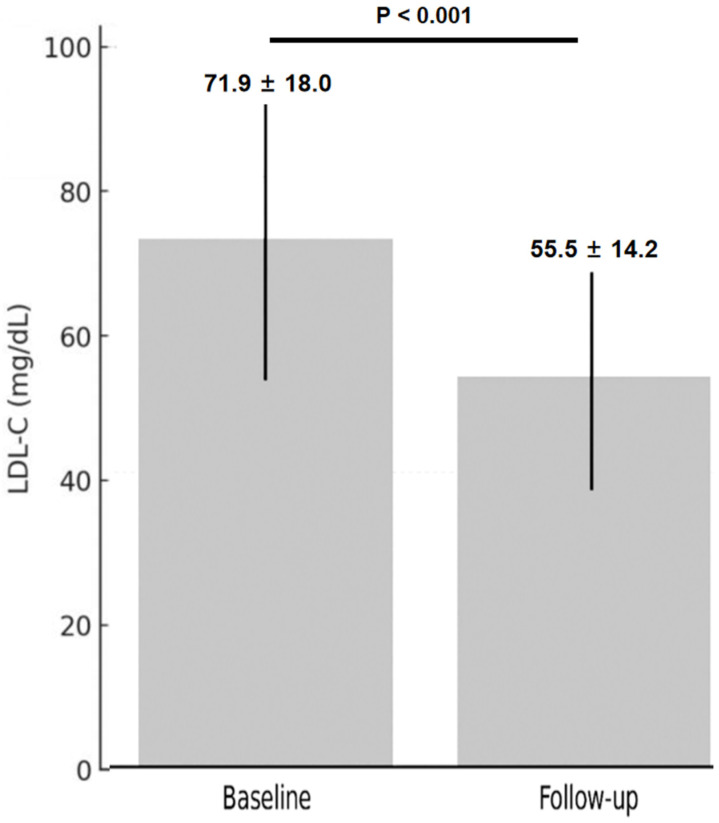
Changes in LDL-C from baseline to follow-up. Mean LDL-C levels at on-treatment baseline and at follow-up after the addition of bempedoic acid (BA), with error bars representing standard deviation, and the corresponding absolute and relative changes.

The addition of BA resulted in a significant reduction in LDL-C levels, from 71.90 ± 18.03 mg/dL to 55.54 ± 14.23 mg/dL (absolute reduction: 16.36 mg/dL; relative reduction: −22.75%; *p* < 0.001) (Table 2, Figure 2).

Values are expressed as mean (SD). Absolute and relative changes were calculated as the difference between baseline and follow-up measurements. LDL-C, low-density lipoprotein cholesterol; HDL-C, high-density lipoprotein cholesterol; eGFR, estimated glomerular filtration rate.

Total cholesterol and non–HDL-C levels decreased by 13.8% and 18.8%, respectively. Triglyceride levels and fasting glucose remained stable throughout follow-up. HDL-C showed a modest decrease (−4.6%).

At follow-up, 48.3% of patients achieved LDL-C levels < 55 mg/dL. When considering untreated baseline LDL-C values, the cumulative LDL-C reduction achieved through sequential lipid-lowering intensification reached 67.5%.

Regarding Lp(a), baseline levels showed a median of 56.8 mg/dL (IQR 78.1), with a wide range (1.8–257.2 mg/dL). More than half of the cohort (56.3%) exhibited Lp(a) levels > 50 mg/dL, indicating a high prevalence of residual cardiovascular risk. Post-treatment measurements were not performed.

### 3.3. Interindividual Variability (Table 3)

No significant differences in LDL-C reduction were observed between patients treated with rosuvastatin- versus atorvastatin-based regimens (14.30 ± 17.90 mg/dL vs. 18.92 ± 22.52 mg/dL; *p* = 0.27). Among statin-tolerant subgroups, patients on background rosuvastatin plus ezetimibe and those on atorvastatin plus ezetimibe exhibited consistent relative reductions in LDL-C (Table 3).

**Table 3:** Relative LDL-C reduction following Bempedoic Acid initiation according to background lipid-lowering therapy categories (*n* = 118).

Background Therapy	*n* (%)	Relative LDL-C Reduction, %	Patient Profile
Rosuvastatin + ezetimibe	76 (64.4)	20.3	Statin-tolerant
Atorvastatin + ezetimibe	38 (32.2)	25.2	Statin-tolerant
PCSK9 inhibitor + ezetimibe	4 (3.4)	42.8	Statin-intolerant
Overall (follow-up)	118 (100)	22.8	—

In a small, descriptive subgroup analysis, patients previously treated with PCSK9 inhibitors (*n* = 4) showed a reduction in LDL-C of 42.8% after BA initiation. Although this finding might suggest a potentially relevant response in this specific combination, the very limited sample size precludes definitive conclusions. Consequently, these results must be interpreted with extreme caution and are considered purely exploratory.

Approximately 13–14% of patients showed minimal or no LDL-C reduction, highlighting clinically relevant interindividual variability in response to ACLY inhibition.

Regarding sex-specific responses, no significant differences were observed in absolute LDL-C reduction between men and women (15.6 ± 19.5 mg/dL vs. 19.6 ± 19.9 mg/dL, respectively; *p* = 0.393). This consistency in BA effectiveness was maintained across background therapy subgroups, with no significant sex-based differences among patients receiving rosuvastatin (*p* = 0.428) or atorvastatin (*p* = 0.577). Although the absolute reduction was numerically higher in the female subgroup, the predictive value of baseline LDL-C and diabetes remained the primary factors associated with of response regardless of sex.

Data are presented as *n* (%) unless otherwise indicated. Relative LDL-C reduction refers to the percentage change from baseline to follow-up. PCSK9 indicates proprotein convertase subtilisin/kexin type 9.

### 3.4. Metabolic and Renal Effects

Treatment with BA was associated with an increase in serum uric acid levels (+0.96 mg/dL, *p* < 0.001), without clinical gout flares.

Serum creatinine increased modestly, while eGFR decreased by approximately 6%. Although these changes were not associated with clinically evident renal impairment during follow-up, no longitudinal or post-treatment data were available to confirm their reversibility.

A subgroup analysis based on baseline renal function revealed no significant differences in eGFR reduction between patients with pre-existing CKD (eGFR < 60 mL/min/1.73 m^2^; *n* = 15) and those with preserved function (−5.60 ± 10.5 vs. −4.67 ± 9.4 mL/min/1.73 m^2^, respectively; *p* = 0.728). The stability of this effect across different baseline eGFR levels reinforces the safety profile of BA in this real-world population.

### 3.5. Factors Associated with LDL-C Reduction

In multivariable linear regression analysis, higher pre-treatment LDL-C levels were independently associated with greater absolute LDL-C reduction following BA initiation (β = −0.515; *p* = 0.001).

In addition, the presence of diabetes mellitus was associated with a significantly larger LDL-C reduction (B = 13.8 mg/dL; *p* = 0.024).

These findings suggest that patients with a higher baseline lipid burden or underlying metabolic dysregulation may derive greater benefit from ACLY inhibition.

Multivariable analyses were supplemented by sex-stratified correlations to ensure consistency. Baseline LDL-C remained a powerful independent predictor of absolute reduction in both men (r = −0.695, *p* < 0.001) and women (r = −0.815, *p* < 0.001). These results indicate that while women were numerically fewer in the cohort, the underlying metabolic response to ACLY inhibition follows the same predictive pattern as in men.

### 3.6. Factors Associated with LDL-C Target Attainment

Binary logistic regression analysis identified higher pre-treatment LDL-C levels (*p* = 0.026) and baseline uric acid concentrations (*p* = 0.026) as independent predictors of achieving LDL-C < 55 mg/dL.

These results indicate that both baseline lipid burden and metabolic profile may influence the probability of achieving guideline-recommended LDL-C targets following BA therapy.

### 3.7. Safety, Adherence, and Treatment Tolerability

The treatment persistence rate was high, with 94.4% of patients completing follow-up.

Three patients (2.4%) discontinued BA due to adverse events, including myalgias (*n* = 1), gastrointestinal symptoms (*n* = 1), and non-specific malaise (*n* = 1). No cases of gout were reported.

Overall, BA demonstrated a favorable safety and tolerability profile in this real-world, multicentre cohort from Cáceres province.

## 4. Discussion

The present prospective, multicentre study demonstrates that adding BA to intensive background lipid-lowering therapy in a real-world Spanish cohort of CCS patients leads to a statistically significant and clinically meaningful reduction in LDL-C levels of 22.8%, enabling nearly half (48.3%) of the patients to achieve the stringent guideline-recommended target of less than 55 mg/dL. Furthermore, our multivariable analyses identified higher baseline LDL-C levels and the presence of type 2 diabetes mellitus as independent factors associated with a greater absolute LDL-C reduction, suggesting that individuals with a higher metabolic or lipid burden derive the most pronounced benefit from ATP citrate lyase inhibition in clinical practice.

The magnitude of LDL-C reduction observed in our cohort is consistent with randomized clinical trials from the CLEAR program, which demonstrated significant LDL-C lowering with BA across different patient populations [10,11,12]. Moreover, outcome trials have confirmed that BA reduces major adverse cardiovascular events, particularly in statin-intolerant patients [13], and cardiovascular benefit appears to be proportional to the magnitude of LDL-C reduction [14], which is consistent with the well-established LDL-C hypothesis linking LDL-C reduction to cardiovascular risk reduction [2].

Importantly, our findings are consistent with real-world evidence. Recent real-world multicentre studies conducted in Southern Europe have reported LDL-C reductions of approximately 25–30% and target attainment rates exceeding 50% in heterogeneous dyslipidaemic populations. These concordant results support the external validity and reproducibility of BA effectiveness in routine clinical practice [15,16].

However, our study extends these observations by providing additional clinically actionable insights. First, we identified baseline factors associated with LDL-C response, demonstrating that higher baseline LDL-C levels and the presence of diabetes mellitus are associated with greater lipid-lowering effectiveness. This finding supports a more individualized approach to therapy, allowing clinicians to identify patients most likely to benefit from BA. Second, our study highlights a high prevalence of elevated Lp(a), indicating a substantial burden of residual genetically mediated cardiovascular risk not addressed by conventional therapies. Third, the longer follow-up duration compared with previous real-world studies provides a more robust assessment of treatment persistence and metabolic safety, supporting the use of BA as a sustainable long-term strategy.

The high prevalence of elevated Lp(a) observed in our cohort underscores the importance of residual cardiovascular risk beyond LDL-C. As Lp(a) levels are largely genetically determined and minimally influenced by current lipid-lowering therapies, patients with elevated levels may remain at increased risk despite achieving LDL-C targets. However, as Lp(a) was assessed only at baseline and no follow-up measurements were available, these findings should be considered purely descriptive and do not allow any conclusions regarding the effect of BA on Lp(a)-related risk. In this context, further LDL-C reduction with BA may help reduce overall atherogenic burden, even if Lp(a)-related risk persists [19,20]. The relatively high prevalence of elevated Lp(a) in our study likely reflects the highly selected nature of the study population, composed exclusively of patients with CCS who remained above guideline-recommended LDL-C targets despite intensive lipid-lowering therapy. Such patients are expected to carry a greater burden of genetically determined residual cardiovascular risk than unselected ASCVD populations.

From a mechanistic perspective, BA inhibits ACLY, reducing hepatic cholesterol synthesis and enhancing LDL receptor activity [8,9]. However, the variability in LDL-C response observed in our cohort suggests that individual metabolic and genetic factors may influence treatment response [21], as described with other lipid-lowering therapies [22].

The safety profile observed in our study is consistent with previous evidence. BA was generally well tolerated, with a modest increase in serum uric acid levels but no clinical gout events, in line with pooled safety analyses [23]. The observation of baseline uric acid as a possible predictor of response in our multivariable model is an intriguing finding. Mechanistically, it may serve as a surrogate marker for metabolic profiles with higher ACLY activity. However, given the exploratory nature of this study and the lack of correction for multiple comparisons, this association should be interpreted with caution and requires validation in larger cohorts. Importantly, no major safety concerns were identified, and treatment persistence was high, which is essential for long-term cardiovascular risk reduction [24].

Regarding renal safety, our study observed a mean eGFR reduction of 6.0%. While we did not perform a post-treatment washout measurement to empirically confirm reversibility, the potential reversibility of these changes cannot be confirmed in our study and should be interpreted with caution. However, the consistency of eGFR changes across renal function subgroups is a relevant safety observation. In our cohort, patients with baseline renal impairment did not experience a more pronounced decline than those with normal function (−5.60 vs. −4.67 mL/min/1.73 m^2^; *p* = 0.728). This ‘non-progressive’ behavior is consistent with the known mechanism of drugs that interfere with creatinine tubular secretion (via OAT2) rather than glomerular filtration, providing clinical reassurance of its safety in patients with CKD. This mechanism is well-documented in the CLEAR clinical trial program [13], where the slight rise in creatinine reached a plateau early and remained stable throughout the treatment period.

From a clinical perspective, our findings support the positioning of BA as an intermediate therapeutic step in lipid-lowering strategies. In patients with CCS who fail to achieve LDL-C targets despite maximally tolerated statins and ezetimibe, BA provides an effective oral option before escalation to injectable lipid-lowering therapies, including PCSK9 monoclonal antibodies and inclisiran, particularly in healthcare settings with limited access to these therapies. This stepwise approach is aligned with contemporary expert recommendations [17,18] and may improve treatment efficiency and goal attainment. Furthermore, our results are consistent with emerging evidence supporting early combination and triple lipid-lowering strategies to achieve rapid LDL-C control in high-risk patients [25]. In our cohort, a small subgroup of statin-intolerant patients (*n* = 4) already treated with PCSK9 inhibitors (PCSK9i) and ezetimibe showed a further 42.8% reduction in LDL-C upon adding BA. The clinical rationale for this combination is based on complementary mechanisms: BA increases LDL receptor (LDLR) expression by inhibiting hepatic cholesterol synthesis, while PCSK9i prevents the degradation of these same receptors [26]. Furthermore, as BA does not increase PCSK9 levels—a phenomenon often observed with statins—the inhibition of PCSK9 may actually potentiate the effects of BA on LDLR availability. Our real-world observation aligns with phase 2 data showing that adding BA to background PCSK9i therapy can lead to an additional 30% reduction in LDL-C with a safety profile comparable to placebo [26], supporting this ‘triple non-statin’ approach for patients far from target. Real-world implementation of lipid-lowering therapy remains suboptimal. Previous studies have shown that a large proportion of high-risk patients do not achieve LDL-C targets despite available treatments [4,5]. This persistent gap highlights the importance of practical and accessible therapies such as BA, which can be easily incorporated into routine clinical practice.

Although our study did not include a formal pharmacoeconomic analysis, previous real-world data suggest that incorporating BA before PCSK9 inhibitors may reduce overall healthcare costs while maintaining effective LDL-C control [14], supporting its role in cost-conscious treatment strategies.

An important consideration in our cohort is the sex distribution, as 79.2% of the participants were male. Sex and gender are increasingly recognized as independent determinants of lipid metabolism and cardiovascular risk profiles. In CCS, women often present different pathophysiological phenotypes, frequently involving a supply-demand imbalance rather than traditional plaque rupture. These differences remain underappreciated in clinical practice and may influence therapeutic responses and outcomes [27]. In our sub-analysis, the predictive value of baseline LDL-C and diabetes for BA response appeared consistent across sexes; however, the lower representation of women in this real-world registry may limit the generalizability of these findings to the female population.

Several limitations should be acknowledged. First, its multicentre observational and non-comparative design lacks a control group, which limits the ability to establish direct causality. Although we ensured stable background lipid-lowering therapy and monitored treatment persistence through clinical interviews and pharmacy records, we cannot entirely exclude the influence of unmeasured factors such as lifestyle modifications. The relatively small sample size restricts the statistical power of subgroup analyses, and the absence of molecular biomarkers precludes deeper mechanistic insights. Second, Lp(a) levels were assessed only at baseline to describe the cohort’s residual risk profile; because serial post-treatment measurements were not performed, this lack of follow-up data must be recognized as a study limitation, as no conclusions can be drawn regarding the longitudinal effect of BA on Lp(a) levels or its associated risk. In addition, although the follow-up duration was longer than that reported in some previous real-world studies, it remains relatively short for evaluating long-term cardiovascular outcomes, treatment persistence, and the durability of the lipid-lowering response. Consequently, no conclusions can be drawn regarding the effect of BA on long-term cardiovascular events. The underrepresentation of women (20.8%) is a significant limitation. While this reflects the consecutive recruitment in our clinical setting, it may affect the generalizability of the results regarding sex-specific metabolic responses to ACLY inhibition. Finally, our exploratory analysis was not applied alongside an alpha-correction for multiple comparisons, which may increase the risk of Type I error for secondary factors associated with target attainment, such as uric acid. Additionally, no formal internal validation was performed, and therefore these findings should be considered exploratory.

Despite these limitations, the prospective design, inclusion of consecutive patients, and comprehensive metabolic evaluation provide robust real-world evidence supporting the clinical use of BA in patients with CCS. Our findings reinforce the importance of individualized treatment strategies and support the integration of BA into contemporary lipid-lowering algorithms.

These results reinforce the strategic role of BA in clinical practice, offering a personalized approach to treatment intensification before considering more resource-intensive therapies.

## 5. Conclusions

In CCS patients not achieving LDL-C targets despite intensive therapy, ACLY inhibition with BA provides a clinically meaningful reduction in LDL-C with a favorable safety profile. These findings support its role as an effective intermediate step in lipid-lowering strategies, particularly in patients requiring additional LDL-C reduction or with statin intolerance.

## Figures and Tables

**Table 1:** Baseline Clinical Characteristics of the Study Cohort (*n* = 125).

Characteristics	Value
Demographics	
Age, years, mean ± SD	62.4 ± 10.1
Age at onset of coronary disease, years	57.2 ± 10.1
Male sex, *n* (%)	99 (79.2)
BMI, kg/m^2^, mean ± SD	28.1 ± 4.0
Waist circumference, cm, mean ± SD	101.6 ± 13.8
Cardiovascular risk factors	
Hypertension, *n* (%)	67 (53.6)
Type 2 diabetes mellitus, *n* (%)	28 (22.4)
Dyslipidaemia, *n* (%)	108 (86.4)
Current smoking, *n* (%)	20 (16.0)
Obesity (BMI ≥ 30 kg/m^2^), *n* (%)	40 (32.0)
Family history of premature ASCVD, *n* (%)	62 (49.6)
Comorbidities	
Peripheral arterial disease, *n* (%)	9 (7.2)
Cerebrovascular disease, *n* (%)	3 (2.4)
eGFR < 60 mL/min/1.73 m^2^, *n* (%)	25 (20.0)
Atrial fibrillation, *n* (%)	3 (2.4)
Coronary disease characteristics	
Previous myocardial infarction, *n* (%)	87 (69,6)
Duration of coronary artery disease, years, median (IQR)	1 (0–6)
Multivessel coronary disease, *n* (%)	61 (48.8)
Recurrent coronary events (≥2), *n* (%)	28 (22.4)
Left ventricular systolic dysfunction, *n* (%)	18 (14.4)
Percutaneous coronary intervention (PCI), *n* (%)	107 (85.6)
Coronary artery bypass grafting (CABG), *n* (%)	19 (15.2)
Baseline maximally tolerated lipid-lowering therapy	
Rosuvastatin 20 mg/day, *n* (%)	80 (64.0)
Atorvastatin 80 mg/day, *n* (%)	40 (32.0)
Ezetimibe 10 mg/day, *n* (%)	125 (100.0)
PCSK9 inhibitor (statin intolerance), *n* (%)	5 (4.0)

**Table 2:** Changes in lipid parameters and metabolic markers from baseline to follow-up (N = 118).

Variable	Baseline	Follow-Up	Absolute Change	Relative Change, %
LDL-C (mg/dL)	71.9 ± 18.0	55.5 ± 14.2	−16.4	−22.8
Total cholesterol (mg/dL)	143.0 ± 26.1	123.2 ± 19.6	−19.8	−13.8
Non-HDL-C (mg/dL)	94.1 ± 25.4	76.4 ± 16.7	−17.6	−18.8
HDL-C (mg/dL)	49.0 ± 11.0	46.8 ± 11.2	−2.3	−4.6
Triglycerides (mg/dL)	120.9 ± 93.8	110.7 ± 53.7	−10.2	−8.4
Uric acid (mg/dL)	5.16 ± 1.43	6.12 ± 1.66	+1.0	+18.6
Serum creatinine (mg/dL)	0.96 ± 0.31	1.05 ± 0.36	+0.09	+9.4
eGFR (mL/min/1.73 m^2^)	78.4 ± 15.5	73.7 ± 17.8	−4.7	−6.0
Fasting glucose (mg/dL)	106.5 ± 26.8	109.3 ± 38.7	+2.8	+2.6

## Data Availability

The data presented in this study are available on request from the corresponding author due to privacy and ethical restrictions.

## Abbreviations

The following abbreviations are used in this manuscript:

LDL-C	Low-density lipoprotein cholesterol
HDL-C	High-density lipoprotein cholesterol
eGFR	Estimated glomerular filtration rate
PCSK9	Proprotein convertase subtilisin/kexin type 9
SD	Standard deviation
ASCVD	Atherosclerotic cardiovascular disease
Lp(a)	Lipoprotein(a)
CEIm	Research Ethics Committee for Medicinal Products
